# Factors influencing participation and regular attendance in a program combining physical activity and nutritional advice for overweight and obese pregnant women

**DOI:** 10.1186/s12884-024-06648-z

**Published:** 2024-06-28

**Authors:** Sophie Lelorain, Philippe Deruelle, Hélène Béhal, Elise Machet, Marie Thiblet, Christelle Lengagne-Piedbois, Valerie Deken-Delannoy, Marie Pigeyre

**Affiliations:** 1 Department of Psychology, Research Center in Health, Aging and Sport Psychology, University of Lausanne, Lausanne, Switzerland; 2 Univ. Lille, CHU Lille, Environnement Périnatal et Santé, Lille, EA 4489, F-59000 France; 3 Department of Gynecology, Obstetrics and Fertility, University Hospital, Strasbourg, France; 4 Univ. Montpellier, CHU Montpellier, Montpellier, France; 5 Department of Statistics, Evaluation, Economics and Data-Management, CHU Lille, Lille, F- 59000 France; 6 Comité Nord de la Fédération Française d’Éducation Physique et de Gymnastique Volontaire, Lille, France; 7 Department of Medicine, Endocrinology Division, McMaster University, Hamilton, ON Canada; 8 Institut de psychologie (IP), Quartier UNIL-Mouline Bâtiment Géopolis 4214, Lausanne, CH - 1015 Switzerland

**Keywords:** Obesity, Overweight, Pregnancy, Newborn, Physical activity, Nutrition, Well-being, Uptake, Attendance, Retention

## Abstract

**Background:**

Educational programs incorporating physical activity (PA) sessions and nutritional workshops have demonstrated potential benefits for overweight and obese pregnant women. However, participation in such programs remains challenging. This prospective study aimed to investigate the factors influencing participation and regular attendance, while examining changes in health behaviors, along with obstetric and neonatal outcomes.

**Methods:**

Pregnant women with at 12–22 weeks’ gestation a BMI ≥ 25 kg/m2 were invited to join an educational program combining three nutritional workshops conducted in groups and 12 weekly PA sessions. They self-selected their participation into the program. Regardless of program uptake and regularity of attendance, the women’s PA levels, eating behaviors, and affectivity were assessed using validated questionnaires at 20–24 weeks, 32–34 weeks, and postpartum. A multivariable logistic regression model was used to determine the factors influencing participation.

**Results:**

Of the 187 women enrolled in the study, 61.5% agreed to participate in the program. Of these, only 45% attended six or more sessions (regardless of the nature of sessions, i.e. nutritional workshops and/or PA sessions), while only 8.7% attended six or more PA sessions. Participation was associated with higher rates of problematic eating behaviors and lower PA levels at baseline, while regular attendance was mainly associated with higher household incomes. No significant difference was observed between participants and non-participants in terms of changes in eating behaviors, PA levels, or affectivity. However, at the 32–34 week visit, regular participants displayed a higher change in positive affectivity, but unexpectedly also in cognitive restraint, than non-regular participants, a difference that did not persist at postpartum.

**Conclusion:**

The educational program combining nutrition and PA was shown to be safe. Women facing challenges related to health behavior displayed a willingness to sign up for the program, but tailored interventions addressing their individual challenges are needed to improve attendance. Accordingly, four recommendations are proposed for the design of future interventions.

**Trial registration:**

ClinicalTrials.gov; Identifier: NCT02701426; date of first registration: 08/03/2016.

**Supplementary Information:**

The online version contains supplementary material available at 10.1186/s12884-024-06648-z.

## Introduction

Obesity is a major threat to public health and has been listed as the sixth most important risk factor contributing to the overall global burden of disease. In 2022, the World Health Organization estimated that almost 60% of adults in the European region were either overweight or affected by obesity, with almost 23% of adults being classified as obese [1]. These numbers reflect a significant and growing problem in obstetrics [2–4]. Pregnancy is a special time for family health education. A balanced diet accompanied by dietary advice and physical activity (PA) appropriate to pregnancy could help control weight gain in mothers with obesity, and prevent inadequate micronutrient status [2–4]. Recommendations on maternal nutrition aim to improve the quality of individuals diets, notably by reducing excessive intake of simple carbohydrates and fat, increasing the consumption of fiber-rich foods, and restoring the sensations of hunger and satiety to induce more adaptive eating behaviors [5, 6]. However, starting a PA during pregnancy can seem difficult, because pregnancy is often associated with increased fatigue and thus a need to rest. These ideas must be challenged since the practice of reasonable and appropriated PA during pregnancy, such as active walking, gymnastics, or swimming, is safe and provides real benefits for both the mother (e.g., lower cesarean rates, as well as reduced fatigue, lumbar pain, and anxiety) [7–9] and the newborn (e.g., improved memory and learning capacities and greater adaptability to stressful situations) [10]. The American College of Obstetricians and Gynecologists (ACOG) recommends low to moderate intensity PA for all pregnant women for at least 20–30 min a day [11]. Similarly, since 2019, the French National Authority for Health (HAS) has recommended a weekly average of 150–180 min [12].

Previous studies have suggested that maternal obesity during pregnancy could be effectively managed through educational programs offering nutritional advice and appropriate PA [13–15]. If we want women to follow this type of program, thus improving their health and that of their babies, it is important to understand the incentives that influence both participation in the intervention program and regular attendance. Most research into lifestyle intervention programs in overweight or obese pregnant women has aimed to test program effectiveness, but few studies have had as their primary objective to determine the variables associated with program participation and attendance. In a meta-analysis of various studies evaluating interventions based on PA and/or nutritional advice designed to control weight gain in pregnant women, the authors found that such programs reduced gestational weight gain by an average of 1.4 kg in the intervention groups compared to the control groups. Nonetheless, the interventions groups did not differ from the control groups with regard to the proportion of women exceeding the gestational weight recommended by the Institute of Medicine. The authors of the meta-analysis propose that the contrasting results could potentially be explained by the programs’ participation rates, lamenting the paucity of information on participation and attendance given in the studies [16]. Thus, while they conclude that setting up the intervention programs was useful, they also suggest that participation and attendance should be assessed, along with the factors that influence them. We can assume that the sociodemographic variables that usually explain health-related behaviors, such as high incomes, will also explain participation in and attendance of interventions in the specific case of overweight and obese women. Sociodemographic variables will thus be taken as candidate variables. We also took an interest in the medical variables, PA, eating behaviors, and affectivity of the pregnant women as candidate variables, since difficulties in these areas could trigger women to take action or deter them from trying something that seems too challenging given their current condition.

The primary objective of our study was therefore to determine the factors that explain participation and regular attendance in a lifestyle intervention in overweight or obese pregnant women. We thus investigated whether not only sociodemographic, but also medico-behavioral factors might influence participation and regular attendance in a program combining PA and nutritional advice. Our secondary goal was to analyze the evolution of PA, eating behaviors, and affectivity during participation, as well as to examine obstetric and neonatal outcomes according to whether women participated in the program or not.

## Methods

The study adheres to the STROBE guidelines for the reporting of cohort observational studies.

### Type of study and inclusion criteria

We conducted a single-center prospective study on pregnant women, aged 18–45 years old, with singleton pregnancy and a BMI ≥ 25 kg/m^2^, at the University Hospital of Lille in northern France.

### Procedure and ethical authorization

During the antenatal visits, we systematically offered women at their 12th and 22^+ 6th^ week of pregnancy the opportunity to participate in a program combining nutritional workshops and PA sessions. Our intervention was presented as an educational program called “Eat well, move well for baby’s health”. Participation in the program was not offered to women with a medical condition that could interfere with PA: i.e., a history of more than two miscarriages, severe heart disease (arrhythmia, a history of myocardial infarction), first trimester bleeding, multiple pregnancy, unstable thyroid disease, pre-existing hypertension, or diabetes.

The women could decide whether or not to participate in this program, thus self-selecting into a participant or non-participant group. In both cases, they were asked whether they agreed to be included in a study aiming to evaluate the factors that influence program participation and to collect data on pregnancy, delivery, neonatal and postpartum outcomes (See additional file 1 for an overview of the study). The women were provided with complete oral and written information. Signed informed consent was collected from each women before they joined the study. The protocol was approved by the « Comité de Protection des Personnes Nord-Ouest IV (Ethics Committee) » (2015-A01085-44). This study was registered on the site ClinicalTrial.gov (no. NCT02701426). The analysis was performed on all women who maintained their consent until the end of the study, including patients who did not complete the entire program.

### Description of the educational program

The program “Eat well, move well for baby’s health” took place between weeks 24 and 36 of pregnancy. In terms of nutritional support, participants were asked to participate in three workshops in groups of 10–15 participants. The workshops lasted two hours and were spread over three months (i.e., one workshop per month) following the initial assessment. These workshops aimed to inform the women about the nutritional guidelines for pregnancy and gestational weight gain, both of which were adapted to overweight and obese women. The workshops took place in the therapeutic kitchen of the hospital’s maternity ward, which made it possible to hold culinary workshops. The detailed contents of the three nutritional workshops are presented in the additional file 2.

In terms of PA, the program (adapted to pregnancy) included sessions developed by the Northern Committee of the French Federation of Physical Education and Voluntary Gymnastics (EPGV) with aerobics and gentle muscle strengthening. The program lasted 12 weeks per patient. Three weekly slots were proposed in the maternity ward with the schedules adapted to working hours. The women were asked to attend at least one session per week, and were strongly encouraged to do second and third sessions on their own, outside the maternity ward. The additional sessions could include an active walk, an indoor gym class in a club, an aqua gym session, or even home exercises suggested by the sports coach. Each session was limited to 10–12 patients to allow the coach to provide personalized advice. Gradually, the patients were given further encouragement to increase their activity. They could note and track their progress using a logbook.

### Measures and assessments

Three questionnaires to assess eating behavior, PA, and affectivity were given to the women during the visits at 20–24 weeks, 32–34 weeks, and postpartum visit (6–8 weeks after delivery).

*Eating behavior* was assessed using the TFEQ (18 items) validated in French and tested on pregnant women [17–19]. Three factors relating to eating behavior factors were assessed in this questionnaire: cognitive restraint (CR), i.e., the conscious effort to restrict food intake to control body weight, emotional eating (EE), i.e., the tendency to eat in response to negative emotions, and uncontrolled eating (UE), i.e., the tendency to overeat accompanied by a loss of control over food consumption.

*Physical activity* was assessed using the Pregnancy Physical Activity Questionnaire (PPAQ) [20]. This self-administered questionnaire with 33 questions provided a qualitative (activity type) and quantitative view of the activity. An intensity was assigned to each activity using the Metabolic Equivalents (MET) table. The MET is a unit used to estimate the metabolic cost of PA: one MET is approximately equal to a person’s resting energy expenditure. The time devoted to each activity, as reported by the women themselves, was then multiplied by the corresponding intensity to obtain the average energy expenditure per week (MET.hours/week). The activities were classified into five categories by type: household/care (13 activities), occupational (five activities), transportation (three activities), sports/exercises (seven activities plus two open-ended questions), and inactivity (three activities). In addition, each activity was assigned to one of four categories based on its intensity: sedentary (< 1.5 METs), light (1.5–2.9 METs), moderate (3.0–6.0 METs), and vigorous (> 6.0 METs). The variable used as a participation factor was the total number of METs per hour of PA per week per patient, defined as total PA (MET.h/week). The volume of total PA corresponded was considered light activity if the score was < 600 MET.h/week, moderate if the score was between 600 and 1,500 MET.h/week, and intense if the score was > 1,500 MET.h/week.

*Affectivity* was assessed using the PANAS questionnaire, which is sensitive to changes over time, and is intended to measure mood through positive and negative affectivity. The PANAS is validated in French and has been used in pregnant women [21–24].

To achieve the primary goal, which was to study the variables influencing program participation, we analyzed 12 *a priori* candidate variables: age, pre-gestational BMI, comorbidities (hypertension, history of cesarean section, and early gestational diabetes detected in the first trimester), parity, socio-professional category, income, smoking, and TFEQ (three dimensions), and PPAQ and PANAS (two dimensions) scores.

To achieve the secondary goals, namely to analyze the development of diet, PA, and affectivity during the program and to examine obstetric and neonatal outcomes in light of participation and attendance status, we defined *attendance* as the number of PA and nutritional sessions attended. Women were classified as “regular” when they attended six or more sessions (e.g., three nutritional workshops + four PA sessions) and “non-regular” when they attended five or fewer sessions.

### Sample size

In the LIMIT study [25], which is similar to ours, 5474 women were invited to participate and 2212 (40%) accepted. Our main objective is to study the factors associated with participation. This requires multivariate analysis, which requires at least 10 events per explanatory variable [26]. Therefore, we need 150 participating patients to study 15 factors. Due to the expected imbalance between the groups (40% participants vs. 60% non-participants), a total of 375 women are needed (150 participants and 225 non-participants).

### Statistical analyses

Categorical variables were expressed as numbers (percentage). Quantitative variables were expressed as the mean (standard deviation, SD) or as the median (interquartile range, IQR) for non-Gaussian distribution. The normality of distributions was assessed using histograms and the Shapiro-Wilk test. We began by assessing the determinants of participation in the educational program in bivariate analyses using Student’s t-test or Mann-Whitney U-test according to the distribution of quantitative determinants, using the Chi-square test (or the Fisher’s exact test if cell frequency < 5) for categorical variables and the Chi-square trend test for ordinal variables. Determinants associated with participation at the level of 0.10 in bivariate analyses were introduced into a multivariable logistic regression model using Firth’s penalized likelihood approach to account for the smaller number of patients. Collinearity among candidate variables was examined by calculating the variance inflation factor (VIF). The odds ratios (ORs) of participants vs. non-participants, along with the 95% confidence intervals (CIs), were estimated as the effect sizes. The same methodology was used to identify determinants of regular attendance in the educational program among participants. Due to collinearity between socio-professional status, personal income, and household income, household income was selected as a candidate variable in the multivariable model.

Obstetrical and neonatal outcomes were compared according to participation using the Student’s t-test or Mann-Whitney U-test according to the distribution of quantitative outcomes and using the Chi-square test (or Fisher’s exact test) for binary outcomes.

The development of the health-related behavior parameters over time was compared between participants and non-participants using a longitudinal analysis of covariance (ANCOVA) taking into account the correlation between the repeated measures within the same subject. A linear mixed model (unstructured covariance pattern model) of the change recorded at the follow-up visit (32–34 weeks and postpartum) from baseline (20–24 weeks) for each behavior parameter was created by including participation status, time (as two-level categorical variable), and the interaction term between participation status and time as fixed effects. In this model, the baseline value of the studied behavior parameter, age, pre-gestational BMI, and educational level were considered pre-specified covariables. The adjusted mean differences in change from baseline between participants and non-participants, as calculated from LSMEANS values, are reported as effect sizes. Statistical testing was done at the two-tailed α-level of 0.05. No statistical comparisons were made for categorical variables with a frequency < 8 in the overall sample. The data were analyzed using the SAS software version 9.4 (SAS Institute, Cary, NC).

## Results

### Sample

Participants were recruited and followed up between February 2016 and October 2018. A total of 195 women consented to enroll in our study. Seven withdrew their consent during the study period and one underwent a termination of pregnancy at 17 weeks, resulting in 187 women being included in the study.

### Main goal: factors influencing participation in the educational program

Of the 187 women included in the study, 115 (61.5%) agreed to participate in the educational program and 72 (38.5%) declined to participate.

Socio-demographic, medical, and behavioral factors known at baseline were compared between participants and non-participants in bivariate analyses (Table 1). None of the socio-demographic or medical variables explained participation in the study, but in the case of medical variables, it should be noted that the overall proportions of comorbidities, such as prior hypertension and early gestational diabetes were low (< 10%). Among health-related behaviors, problematic eating behaviors (TFEQ) were significantly higher in participants compared to non-participants.

**Table 1:** Baseline factors influencing participation in the educational program

	*N*	Participants *n* = 115	*N*	Non-participants *n* = 72	*p*-value
**Socio-demographic status**					
Age	115	29.7 ± 5.1	72	28.8 ± 4.7	0.26
Socio-professional category	115		71		0.066
Unemployed/unskilled manual worker		40 (34.8)		21 (29.6)	
Skilled manual worker		17 (14.8)		22 (31.0)	
Skilled non-manual worker		42 (36.5)		19 (26.8)	
Intellectual/managerial profession		16 (13.9)		9 (12.7)	
Educational level	115		71		0.67
< NVQ level 1,2		7 (6.1)		1 (1.4)	
High school graduate		32 (27.8)		29 (40.9)	
> High school graduate		76 (66.1)		41 (57.7)	
Personal income per month	115		70		0.34
<€763		27 (23.5)		14 (20.0)	
€763–€1,265		31 (27.0)		24 (34.3)	
€1,266–€1,905		36 (31.3)		25 (35.7)	
€1,905–€2,600		13 (11.3)		7 (10.0)	
>€2,600		8 (7.0)		0 (0)	
Household income per month	115		70		0.28
<€763		7 (6.1)		2 (2.9)	
€763–€1,265		15 (13.0)		5 (7.1)	
€1,266–€1,905		16 (13.9)		11 (15.7)	
€1,905–€2,600		29 (25.2)		23 (32.9)	
>€2,600		48 (41.7)		29 (41.4)	
**Medical history**					
Number of nullipara	115	69 (60.0)	72	36 (50.0)	0.18
Body mass index	114	30.0 [27.5–32.8]	72	30.0 [26.9–32.4]	0.63
History of hypertension	115	2 (1.7)	72	2 (2.8)	NA
History of c-section	115	8 (7.0)	72	7 (9.7)	0.50
Early gestational diabetes	115	11 (9.6)	72	5 (6.9)	0.53
**Health-related behaviors**					
Smoking (ever smoked / no)	115	11 (9.6)	72	8 (11.1)	0.73
TFEQ scores	112		72		
Cognitive restraint		39.8 ± 17.2		32.6 ± 18.6	0.009
Uncontrolled eating		35.0 ± 18.1		27.0 ± 17.7	0.004
Emotional eating		43.8 ± 23.7		30.2 ± 22.8	< 0.001
PPAQ (score in MET.h/week)	111	227.9 [161.7-293.8]	72	240.5 [185.2-335.3]	0.084
PANAS	112		71		
Positive affectivity		34.0 ± 6.1		33.3 ± 6.5	0.49
Negative affectivity		21.5 ± 7.0		19.8 ± 6.1	0.11

Values expressed as numbers (%), mean ± SD or median (IQR);

Abbreviations SD = Standard Deviation; IQR = Interquartile Range, NA = Not Applicable, TFEQ = Three Eating Questionnaire scores; PPAQ = Pregnancy Physical Activity Questionnaire; PANAS = Positive and Negative Affect Schedule (PANAS-SF). MET = Metabolic Equivalent of Task

In the multivariable model (Table 2), only cognitive restraint and PA were significantly associated with participation: greater cognitive restraint was associated with participation, OR = 1.02, 95% CI (1.00–1.04), whereas a higher PA was associated with non-participation, OR = 0.97, 95% CI (0.95–0.99).

**Table 2:** Multivariable model of factors influencing participation in the educational program

	OR (95%CI)*	*p*-value
**Socio-professional category**		0.67
Unemployed/unskilled manual worker	1.00 (ref)	
Skilled manual worker	0.63 (0.25–1.61)	
Skilled non-manual worker	1.13 (0.50–2.53)	
Intellectual/managerial profession	0.92 (0.32–2.66)	
**TFEQ scores**		
Cognitive restraint	1.02 (1.00–1.04)	0.029
Emotional eating	1.02 (0.99–1.03)	0.066
Uncontrolled eating	1.02 (0.99–1.04)	0.15
PPAQ (score in MET.h/week), per 10-unit increase	0.97 (0.95–0.99)	0.045

Abbreviations OR: Odds Ratio, 95%CI: 95% Confidence interval, TFEQ = Three Eating Questionnaire scores, PPAQ = Pregnancy Physical Activity Questionnaire, MET = Metabolic Equivalent of Task

*OR are expressed for a one-unit increase unless otherwise indicated and estimated in favor of participation in the educational program

### Main goal: factors influencing attendance in the educational program

For two participants, the information about regularity was missing. Only 51 (45%) of the participants were classified as regular. The median attendance percentage for the nutritional workshops was 66.7% [IQR 0-100] and 8.3% [IQR 0-66.7] at PA sessions. A total of 34 participants (30%) did not attend any of the three nutritional workshops, while 46 (41%) did not take part in any PA session. By contrast, 19 participants (17%) participated in 12 or more sessions, including five participants (4%) who took part in more than 20 sessions.

As shown in Table 3, in bivariate analyses, age, educational level, and incomes were significantly higher for regular attendees compared to non-regular attendees. No differences were found in medical variables between the two groups. With regard to behavioral and psychological variables, regular participants had lower rates of uncontrolled eating (*p* = 0.049), higher positive affectivity (*p* = 0.003), and lower negative affectivity (*p* = 0.023) than non-regular ones.

**Table 3:** Baseline factors influencing educational program attendance

	*N*	Regular *n* = 51	*N*	Non-regular *n* = 62	*p*-value
**Socio-demographic status**					
Age, years	51	31.2 ± 4.7	62	28.4 ± 5.1	0.003
Socio-professional category	51		62		0.074
Unemployed/unskilled manual worker		12 (23.5)		26 (41.9)	
Skilled manual worker		7 (13.7)		10 (16.1)	
Skilled non-manual worker		21 (41.2)		21 (33.9)	
Intellectual/managerial profession		11 (21.6)		5 (8.1)	
Educational level	51		62		0.003
< NVQ level 1,2		0 (0)		6 (9.7)	
High school graduate		10 (19.6)		21 (33.9)	
> High school graduate		41 (80.4)		35 (56.4)	
Personal income	51		62		0.006
<€763		8 (15.7)		18 (29.0)	
€763–€1,265		11 (21.6)		19 (30.7)	
€1,266–€1,905		18 (35.3)		18 (29.0)	
€1,905–€2,600		8 (15.7)		5 (8.1)	
>€2,600		6 (11.8)		2 (3.2)	
Household income	51		62		< 0.001
<€763		0 (0)		7 (11.3)	
€763–€1,265		4 (7.8)		10 (16.1)	
€1,266–€1,905		6 (11.8)		10 (16.1)	
€1,905–€2,600		9 (17.6)		19 (30.6)	
>€2,600		32 (62.7)		16 (25.8)	
**Medical history**					
Number of nullipara	51	27 (52.9)	62	41 (66.1)	0.15
Body mass index	50	29.1 [27.3–31.3]	62	30.1 [27.6–35.1]	0.19
History of hypertension	51	1 (2.0)	62	1 (1.6)	NA
History of c-section	51	4 (7.8)	62	4 (6.4)	1
Early gestational diabetes	51	7 (13.7)	62	4 (6.5)	0.22
**Health-related behaviors**					
Smoking (ever smoked / no)	51	4 (7.8)	62	7 (11.3)	0.75
TFEQ scores	50		60		
Cognitive restraint		41.0 ± 19.5		38.6 ± 15.4	0.48
Uncontrolled eating		30.1 ± 18.4		37.7 ± 17.1	0.049
Emotional eating		42.8 ± 25.9		43.7 ± 21.4	0.84
PPAQ (score in MET.h/week)	50	226.9 [184.5–287.0]	59	236.7 [159.52–308.3]	0.51
PANAS	50		60		
Positive affectivity		35.9 ± 4.6		32.7 ± 6.5	0.003
Negative affectivity		19.7 ± 5.9		22.5 ± 6.8	0.023

Values expressed as numbers (%), mean ± SD or median [IQR];

*Abbreviations* SD = Standard Deviation; IQR = Interquartile Range, NA = Not Applicable, TFEQ = Three Eating Questionnaire scores; PPAQ = Pregnancy Physical Activity Questionnaire; PANAS = Positive and Negative Affect Schedule (PANAS-SF). MET = Metabolic Equivalent of Task

In the multivariable model (Table 4), only household income remained significantly associated with attendance: higher income was associated with regular participation, OR = 1.69, 95% CI (1.07–2.66).

**Table 4:** Multivariable model of factors influencing educational program attendance

	OR (95%CI)*	*p*-value
Age, years	1.06 (0.97–1.17)	0.20
Educational level		0.80
< NVQ level 1,2	1.00 (ref)	
High school graduate	3.36 (0.09–119.45)	
> High school graduate	2.95 (0.09–102.84)	
Household income	1.60 (1.02–2.49)	0.04
**TFEQ scores**		
Uncontrolled eating	1.00 (0.97–1.02)	0.71
**PANAS**		
Positive affectivity	1.05 (0.97–1.14)	0.23
Negative affectivity	0.96 (0.89–1.04)	0.40

*Abbreviations* OR: Odds Ratio, 95%CI: 95% Confidence Interval, TFEQ = Three Eating Questionnaire scores, PPAQ = Pregnancy Physical Activity Questionnaire, MET = Metabolic Equivalent of Task

*OR are expressed for a one-unit increase and estimated in favor of regular attendance

### Secondary goal: comparing the development of health-related behaviors and affectivity between participants and non-participants (Fig. 1; Table 5)

**Fig. 1:**
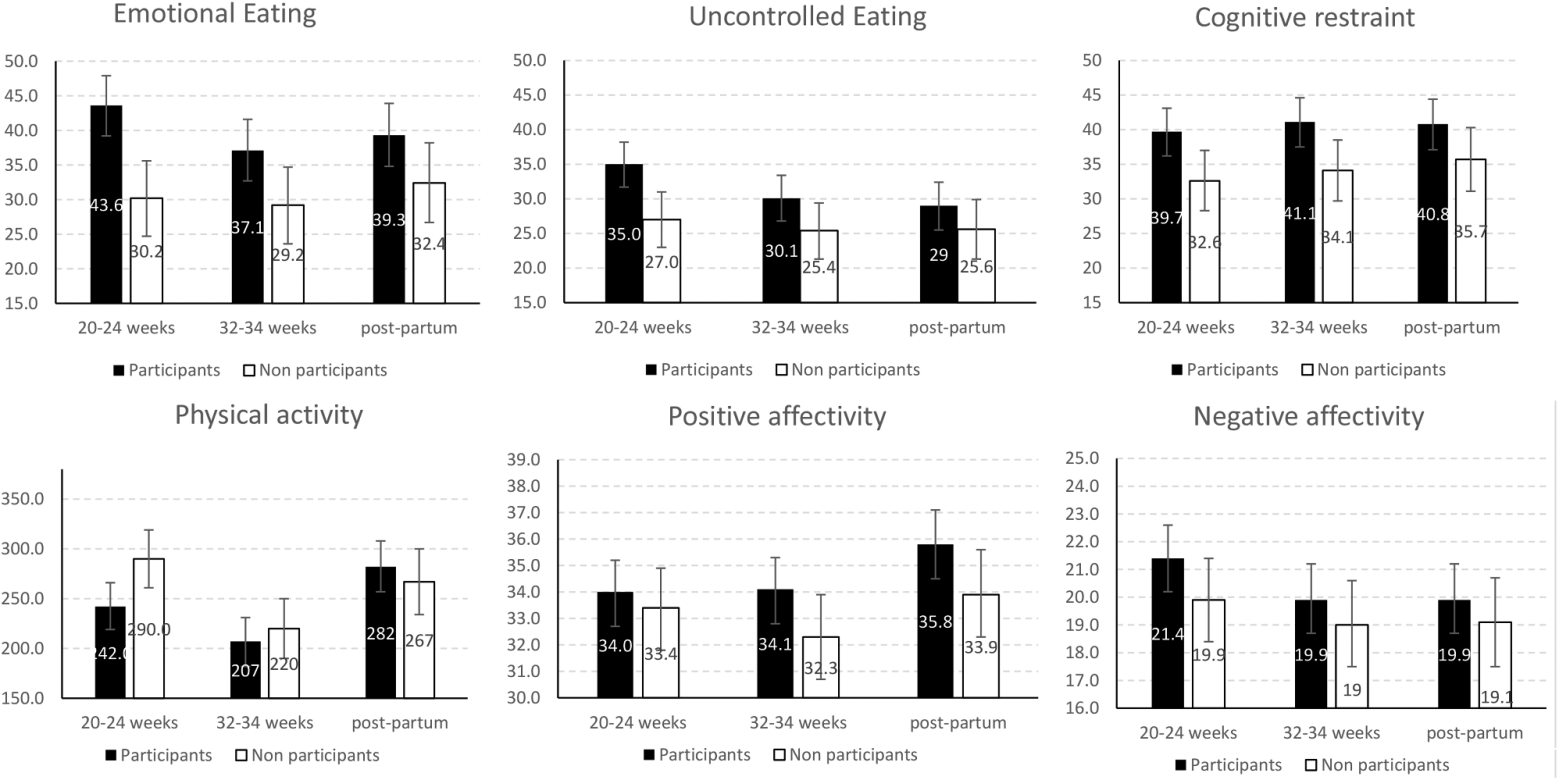
Means of eating behaviors, physical activity (Met.h per week) and affectivity according to *participation*. *Note* Error bars = 95% confidence intervals of the means. Means and 95%CI were estimated using a mixed model, considering variance of the three repeated measure of each outcome

**Table 5:** Development of eating behaviors, physical activity, and affectivity according to participation

	Participants	Non-participants	Difference in change from baseline^#^	
	Mean (95%CI)*	Mean (95%CI)*	Mean (95%CI)	*p*-value
**Uncontrolled eating**				
20–24 weeks (baseline)	35.0 (31.7 to 38.2)	27.0 (23.0 to 31.0)		
32–34 weeks	30.1 (26.8 to 33.4)	25.4 (21.3 to 29.4)		
Post-partum	29.0 (25.5 to 32.4)	25.6 (21.3 to 29.9)		
Change (32–34 weeks – baseline) ^#^	-2.0 (-5.9 to 1.9)	-0.6 (-5.2 to 3.9)	1.4 (-2.6 to 5.3)	0.50
Change (postpartum –baseline) ^#^	-3.4 (-7.6 to 0.9)	-0.7 (-5.7 to 4.3)	2.7 (-2.2 to 7.5)	0.28
**Emotional eating**				
20–24 weeks (baseline)	43.6 (39.2 to 47.9)	30.2 (24.7 to 35.6)		
32–34 weeks	37.1 (32.7 to 41.6)	29.2 (23.6 to 34.7)		
Post-partum	39.3 (34.8 to 43.9)	32.4 (26.7 to 38.2)		
Change (32–34 weeks – baseline) ^#^	-0.8 (-6.0 to 4.3)	1.2 (-4.8 to 7.3)	2.1 (-3.3 to 7.4)	0.44
Change (postpartum –baseline) ^#^	1.5 (-4.0 to 7.0)	4.1 (-2.4 to 10.6)	2.6 (-3.6 to 8.8)	0.40
**Cognitive restraint**				
20–24 weeks (baseline)	39.7 (36.2 to 43.1)	32.6 (28.3 to 37.0)		
32–34 weeks	41.1 (37.5 to 44.6)	34.1 (29.7 to 38.5)		
Postpartum	40.8 (37.1 to 44.4)	35.7 (31.1 to 40.3)		
Change (32–34 weeks – baseline) ^#^	1.8 (-2.3 to 6.0)	-0.1 (-5.0 to 4.8)	-2.0 (-6.3 to 2.4)	0.38
Change (postpartum –baseline) ^#^	1.7 (-2.9 to 6.2)	1.6 (-3.7 to 7.0)	0.0 (-5.3 to 5.2)	0.99
**Physical activity**				
20–24 weeks (baseline)	242 (219 to 266)	290 (261 to 319)		
32–34 weeks	207 (182 to 231)	220 (190 to 250)		
Postpartum	282 (257 to 308)	267 (234 to 300)		
Change (32–34 weeks – baseline) ^#^	**-61 (-90 to -32)**	**-67 (-100 to -33)**	-5.3 (-32.8 to 22.2)	0.70
Change (postpartum – baseline) ^#^	12 (-24 to 48)	-20 (-63 to 23)	-32.0 (-77 to 13)	0.16
**Positive affectivity**				
20–24 weeks (baseline)	34.0 (32.7 to 35.2)	33.4 (31.8 to 34.9)		
32–34 weeks	34.1 (32.8 to 35.3)	32.3 (30.7 to 33.9)		
Postpartum	35.8 (34.5 to 37.1)	33.9 (32.3 to 35.6)		
Change (32–34 weeks – baseline) ^#^	1.0 (-0.5 to 2.5)	0.0 (-1.8 to 1.8)	-1.0 (-2.6 to 0.6)	0.22
Change (postpartum – baseline) ^#^	**2.6 (1.1 to 4.2)**	1.5 (-0.4 to 3.3)	-1.1 (-3.0 to 0.6)	0.19
**Negative affectivity**				
20–24 weeks (baseline)	21.4 (20.2 to 22.6)	19.9 (18.4 to 21.4)		
32–34 weeks	19.9 (18.7 to 21.2)	19.0 (17.5 to 20.6)		
Postpartum	19.9 (18.7 to 21.2)	19.1 (17.5 to 20.7)		
Change (32–34 weeks – baseline) ^#^	-0.9 (-2.4 to 0.5)	-0.6 (-2.3 to 1.1)	0.3 (-1.2 to 1.8)	0.69
Change (postpartum – baseline) ^#^	-0.9 (-2.5 to 0.7)	-0.6 (-2.4 to 1.2)	0.3 (-1.4 to 2.1)	0.71

* Means (95%CI) were estimated using a mixed model, considering variance of the three repeated measure of each outcome

# changes were adjusted for baseline value, age, pre-gestational BMI, and educational level. If the confidence interval does not contain the value of 0, then the change is statistically significant and is in bold

#### Eating behavior

None of the three eating behaviors developed differently between the two groups (participants vs. non-participants).

#### Physical activity

PA *decreased* significantly in each group between baseline and the visit in week 32–34 but without significant differences between the two groups. Nor did the two groups differ in their development between baseline and postpartum.

#### Affectivity

Between the baseline and the postpartum assessment, positive affectivity increased significantly in the participants, but not in the non-participants, although the difference between the two groups was not significant (*p* = 0.19). No difference in negative affectivity was observed between the two groups.

#### To summarize

The development of eating behaviors, physical activity, and affectivity did not differ between participants and non-participants.

### Secondary goal: comparing the development of health-related behaviors and affectivity between regular and non-regular participants (Fig. 2; table 6)

**Fig. 2:**
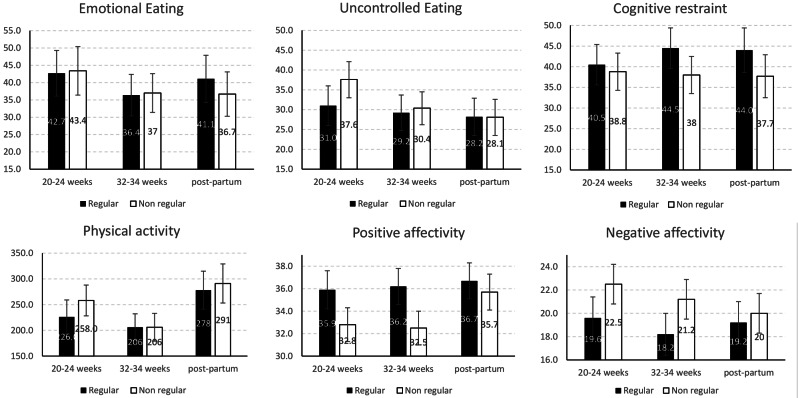
Means of eating behaviors, physical activity (Met.h per week) and affectivity according to *attendance*. *Note* Error bars = 95% confidence intervals of the means. Means and 95%CI were estimated using a mixed model, considering variance of the three repeated measure of each outcome

**Table 6:** Development of eating behaviors, physical activity, and affectivity according to attendance

	Regular	Non-regular	Difference in change from baseline^#^	
	Mean (95%CI)*	Mean (95%CI)*	Mean (95%CI)	*p*-value
**Uncontrolled eating**				
20–24 weeks (baseline)	31.0 (26.0 to 36.0)	37.6 (33.0 to 42.1)		
32–34 weeks	29.2 (24.7 to 33.7)	30.4 (26.2 to 34.5)		
Postpartum	28.2 (23.5 to 32.9)	28.1 (23.5 to 32.6)		
Change (32–34 weeks – baseline) ^#^	-1.8 (-7.0 to 3.4)	-3.2 (-7.6 to 1.2)	-1.4 (-6.4 to 3.6)	0.58
Change (postpartum –baseline) ^#^	-2.8 (-8.7 to 3.1)	-5.0 (-10.4 to 0.3)	-2.2 (-8.6 to 4.2)	0.50
**Emotional eating**				
20–24 weeks (baseline)	42.7 (36.1 to 49.3)	43.4 (37.4 to 49.5)		
32–34 weeks	36.4 (30.4 to 42.4)	37.0 (31.4 to 42.6)		
Postpartum	41.1 (34.3 to 47.9)	36.7 (30.3 to 43.2)		
Change (32–34 weeks – baseline) ^#^	-2.2 (-9.5 to 5.1)	-1.8 (-8.0 to 4.5)	0.4 (-6.6 to 7.5)	0.90
Change (postpartum –baseline) ^#^	2.7 (-5.0 to 10.3)	-2.0 (-8.8 to 4.8)	-4.6 (-12.5 to 3.2)	0.24
**Cognitive restraint**				
20–24 weeks (baseline)	40.5 (35.6 to 45.4)	38.8 (34.3 to 43.3)		
32–34 weeks	44.5 (39.6 to 49.4)	38.0 (33.5 to 42.5)		
Postpartum	44.0 (38.6 to 49.3))	37.7 (32.5 to 42.9)		
Change (32–34 weeks – baseline) ^#^	**5.8 (0.4 to 11.3)**	-0.6 (-5.3 to 4.1)	**-6.4 (-11.8 to -1.0)**	**0.020**
Change (postpartum –baseline) ^#^	5.6 (-0.6 to 11.7)	-0.8 (-6.4 to 4.8)	-6.3 (-13.1 to 0.4)	0.066
**Physical activity**				
20–24 weeks (baseline)	226 (193 to 259)	258 (228 to 288)		
32–34 weeks	206 (180 to 233)	206 (181 to 231)		
Postpartum	278 (241 to 31)	291 (253 to 327)		
Change (32–34 weeks – baseline) ^#^	**-44 (-78 to -10)**	**-61 (-91 to -31)**	-17 (-50 to 15)	0.28
Change (post–partum – baseline) ^#^	26 (-19 to 71)	14 (-28 to 56)	-12 (-65 to 41)	0.66
**Positive affectivity**				
20–24 weeks (baseline)	35.9 (34.2 to 37.5)	32.8 (31.3 to 34.3)		
32–34 weeks	36.2 (34.6 to 37.8)	32.5 (31.0 to 34.0)		
Postpartum	36.7 (35.1 to 38.3)	35.7 (34.1 to 37.3)		
Change (32–34 weeks – baseline) ^#^	**2.8 (0.8 to 4.8)**	-0.4 (-2.1 to 1.3)	**-3.2 (-5.2 to -1.2)**	**0.002**
Change (postpartum – baseline) ^#^	**3.1 (1.0 to 5.1)**	**2.7 (0.8 to 4.5)**	-0.4 (-2.6 to 1.8)	0.72
**Negative affectivity**				
20–24 weeks (baseline)	19.6 (17.8 to 21.4)	22.5 (20.8 to 24.2)		
32–34 weeks	18.2 (16.4 to 19.9)	21.2 (19.5 to 22.8)		
Post-partum	19.2 (17.4 to 20.9)	20.0 (18.3 to 21.7)		
Change (32–34 weeks – baseline) ^#^	-1.8 (-3.7 to 0.2)	0.0 (-1.7 to 1.8)	1.8 (-0.1 to 3.8)	0.060
Change (postpartum – baseline) ^#^	-0.7 (-2.8 to 1.4)	-1.0 (-3.0 to 1.0)	-0.3 (-2.6 to 1.9)	0.77

* Means(95%CI) were estimated using a mixed model considering variance of the three repeated measure of each outcome

# changes were adjusted for baseline value, age, pre-gestational BMI and educational level. If the confidence interval does not contain the value of 0, then the change is statistically significant and is in bold

#### Eating behavior

The two groups differed in how their cognitive restraint developed between baseline and the visit in week 32–34: regular participants increased their cognitive restraint whereas non-regular ones did not, and this difference in evolution was statistically significant (*p* = 0.02).

#### Physical activity

PA decreased significantly in each group between baseline and the visit in week 32–34, albeit without significant differences between the two groups.

#### Affectivity

Positive affectivity increased significantly in regular participants in weeks 32–34 and at postpartum, whereas it increased only at postpartum in non-regular participants. Therefore, the development of positive affectivity between baseline and the weeks 32–34 was statistically different between the two groups (*p* = 0.002), with regular attendees displaying a more favorable development.

#### To summarize

Regular attendees differed from non-regular ones in two respects: they developed greater positive affectivity, but unexpectedly they also developed more cognitive restraint between baseline and the visit in weeks 32–34.

### Secondary goal: the effects of the educational program on maternofetal outcomes

No significant effects related to the educational program were observed in any of the 17 maternofetal outcomes tested when comparing participants to non-participants (Table 7).

**Table 7:** Obstetrical and neonatal outcomes according to participation

	*N*	Participants *n* = 115	*N*	Non participants *n* = 72	*p*-value
Complication	115	43 (37.4)	71	24 (33.8)	0.62
Gestational diabetes	43	24 (55.8)	24	14 (58.3)	0.84
Gestational hypertension	43	4 (9.3)	24	0 (0)	NA
Preeclampsia	43	0 (0)	24	1 (4.2)	NA
Preterm PROM	43	0 (0)	24	1 (4.2)	NA
Premature birth	43	3 (7.0)	24	2 (8.3)	NA
Gestational weight gain (kg)	109	11.6 ± 7.2	70	10.6 ± 5.8	0.58
C-section	113	31 (27.4)	71	15 (21.1)	0.34
Instrumental vaginal delivery	82	20 (24.4)	56	11 (19.6)	0.51
Birth weight (g)	113	3405 ± 516	71	3481 ± 509	0.33
Umbilical pH < 7.10	113	18 (15.9)	70	5 (7.1)	0.081
Apgar score at 1 min < 7	113	4 (3.5)	71	2 (2.8)	NA
Shoulder dystocia	111	4 (3.6)	71	1 (1.4)	NA
Transfer in ICU	113	3 (2.6)	71	2 (2.8)	NA
Breastfeeding	105	65 (61.9)	64	32 (50.0)	
Formula feeding		26 (24.8)		25 (39.1)	0.14
Mixed feeding		14 (13.3)		7 (10.9)	
Post-partum BMI at T3 (kg/m^2^)	98	30.9 [28.8–34.5]	62	30.6 [27.7–33.4]	0.23
Baby’s weight at T3 (g)	104	4894 ± 623	63	4787 ± 775	0.35

Values expressed as numbers (%), mean ± SD or median [IQR]; T3 = 6 to 8 weeks after delivery

*Abbreviations* SD = Standard Deviation; IQR = Interquartile Range, NA = Not Applicable

## Discussion

### Summary of results

The main goal of this study was to determine the factors that explain participation and regular attendance in a lifestyle intervention in pregnant overweight and obese women. The secondary goal was to compare the development of health-related behaviors and affectivity between groups, as well as maternofetal outcomes.

Interestingly, the factors that explained participation were different from those that explained attendance. Participation was driven by problematic health behaviors, such as problematic eating behaviors—especially cognitive restraint—and low levels of PA. Conversely, regular attendance was driven by sociodemographic, behavioral, and affective resources, namely higher levels of education and income, lower rates of uncontrolled eating, and “better” affectivity, with income representing the only explaining variable in the multivariate analysis. With respect to the secondary goal, the only differences found were that regular participants demonstrated both increased positive affectivity and greater cognitive restraint between baseline and the first visit compared to non-regular participants.

### Prevalence of participation and regular attendance

In our study, 61.5% of women agreed to participate in the educational program. This number corresponds to the upper limit of the range of patient participation in self-management programs for chronic diseases, including obesity, which extends from 10 to 60% [27–29]. Concerns for their baby’s health may explain the “high” rate of participation by these women, which was also seen with regard to weight management advice during pregnancy, with 78% attendance at the first appointment [30]. However, only 45% of participants in our study and 41% in the individual program mentioned earlier (30) attended classes regularly. This drop-out rate is problematic, given that long-term positive outcomes depend on high attendance [31, 32]. Attendance at PA sessions was particularly low, with 50% of women attending less than 8.3% of these sessions.

### Factors determining participation and attendance (main goal)

Participation was mainly explained by cognitive restraint and low levels of PA, difficulties that seemed to trigger participation. This suggests that women are aware of their behavioral problems and willing to try something that may help them cope, regardless of their level of education and income. The latter factors did not impact participation, in contrast to the usual results concerning the impact of socio-economic status on health-related behaviors (e.g. [33]). The fact that our program was free may explain why income did not affect the decision to participate. Furthermore, social desirability—i.e., the tendency to behave in a way that conforms to the expectations of others and society as a whole—may also explain the decision to participate, as there are strong expectations that pregnant women will do what is best for their baby’s health. However, once the program started, the difficulties usually associated with lower attendance in scientific literature were also found to reduce attendance in our study, namely a lower educational level [27, 34, 35], younger age [34], emotional difficulties [36], and problematic eating behavior [34]. In our multivariable model, income was the only variable that significantly explained attendance. Women with lower socio-economic status or who belong to ethnic minorities may consider health less of a priority [37]. Moreover, in challenging socio-economic contexts, a lack of time and support from their partners, which are widely recognized as significant barriers to PA [38–40], may also be an issue. These women may not be able to attend three nutrition classes and 12 exercise classes due to a busy and inflexible work schedule. Furthermore, group educational interventions—although in our case they were limited to 12 participants in order to provide as much personalized advice as possible —may be deemed too general and not tailored to specific personal challenges and situations [37]. Finally, women with low socio-economic status may not feel comfortable in educational groups, perhaps due to low health literacy.

### The development of behavioral variables and affectivity between groups (secondary goal)

With the exception of a greater change in PA in the regular participants compared to non-regular ones, the program did not improve any other variables. An increase in cognitive restraint was even seen in regular attendees at 32–34 weeks. Eating behaviors may require more time to change and should thus probably be addressed before pregnancy to improve health outcomes. Indeed, the program did not improve any mother and infant health outcomes, which confirms the results of a recent meta-review showing almost no health benefits to lifestyle interventions in overweight or obese pregnant women [41]. As discussed in the meta-review, pregnancy, which already implies many changes and difficulties, may not, in fact, be the best period to initiate a behavioral change, but may be better suited to consolidating previous changes of habits. Alternatively, such interventions could be better received and more consistently followed if proposed earlier in pregnancy [35, 42], especially as tiredness in the third trimester of pregnancy prevents engagement in PA [40].

### Suggestions to improve future interventions

We propose four ways of improving future interventions: (1) co-construction of the program with patients as partners, (2) flexible modalities of delivering programs using digital tools, (3) the involvement of partners, and (4) the use of behavioral changes techniques.

(1) Co-construction of the interventions with patients as partners [43–45] is required to ensure that the intervention meets patients’ actual needs and is grounded in their lives in some way. In fact, interventions are often designed by highly educated researchers and clinicians who may lack perspective on the challenges encountered by obese women with low socio-economic status or emotional difficulties. Even the participants’ built environment (e.g., parks, transit, walkability, etc.) can influence the efficacy of interventions [46] and should thus be discussed with participants. (2) Employing e-interventions or combining face-to-face and online sessions is a promising approach, as these methods demonstrate higher retention rates [47] and positive outcomes [48]. The use of an app dedicated to the program with multidisciplinary content has also been praised (39), particularly by women living in disadvantaged neighborhoods [49]. Such devices allow frequent contact with participants, which is a success factor in the interventions [42]. (3) As social support is key to successful lifestyle change, partners or next of kin should be involved from the beginning of the program so that they become more aware of their responsibility to help their partner change. (4) To further help patients implement PA and healthy dietary in their daily life, programs of this kind would greatly benefit from sessions focused on behavioral change techniques (BCT). In a systematic review of PA interventions for overweight and obese pregnant women [50], the most-used BCTs in successful interventions were instructions on how to perform a behavior and behavioral practice/rehearsal (two elements that were implemented in our program) along with self-monitoring of behavior (which was only encouraged in our program), and social support, goal setting outcome, and problem solving (which were not addressed in our program). Finally, feasibility studies aiming to assess the acceptability of interventions by participants and attrition rates, should also be carried out before any large-scale rollout [51, 52].

### Limitations and strengths

Contextual data concerning the intervention are lacking. For example, we have no information about the family support received (or not) by women in relation to program attendance and changing health behaviors, whereas evidence shows that the opinion and support of family impacts attendance rates [34, 50]. We also do not know how the program was conducted and perceived by the participants, whereas peer-support and friendliness on the part of the facilitators are also known to contribute to attendance [37]. Nutritional habits were also not evaluated in detail. The use of additional scales may have revealed changes that were not captured by the single measure we used. Due to low attendance, there may be insufficient statistical power to compare regular and non-regular participants. Finally, results on maternofetal outcomes should be read with extreme caution. Indeed, since the study was not designed to test the effectiveness of the program, no randomization or adjustment for confounding variables in analyses related to maternofetal outcomes was carried out. Despite these limitations, some strengths must be highlighted. Even though this was a single-center study, which limits the generalizability of the results, Northern France, where it was conducted, is the region that is most impacted by obesity [53] and thus highly relevant for such a study. Another strength is that we considered both participation and regular attendance. This distinction made it possible to demonstrate that even though problematic health behaviors triggered participation, showing the willingness of women to address their bad habits, low incomes remained strong barriers that must be concretely addressed.

## Conclusion

Our study highlights the complex interplay of factors influencing participation and regular attendance in a lifestyle intervention program for overweight and obese pregnant women. While cognitive restraint and low PA levels were the primary motivators for initial participation, sustained attendance was significantly influenced by socioeconomic factors, particularly income. The lack of significant improvements in the targeted health-related behaviors and maternal and neonatal outcomes suggests that such interventions may need to be started early and to be supplemented by more tailored approaches to address specific barriers faced by lower-income participants. Future programs should consider integrating flexible delivery methods, partner involvement, and behavior change techniques to enhance effectiveness and adherence. The target participants should also be included in the design of the intervention. Understanding these dynamics is crucial for designing more inclusive and impactful health interventions during pregnancy.

### Electronic supplementary material

Below is the link to the electronic supplementary material.

Supplementary Material 1

Supplementary Material 2

Supplementary Material 3

## Data Availability

The datasets generated and analyzed within the framework of the current study are not publicly available as they are the exclusive property of the University Hospital of Lille (CHU de Lille). However, they are available upon reasoned and well-founded request to the corresponding author after authorization by the CHU de Lille.

## References

[1] World Health Organization. WHO European Regional Obesity Report 2022 [Internet]. Copenhagen: WHO Regional Office for Europe. 2022. 220 p. https://iris.who.int/bitstream/handle/10665/353747/9789289057738-eng.pdf?sequence=1.

[2] Margerison Zilko CE, Rehkopf D, Abrams B (2010). Association of maternal gestational weight gain with short- and long-term maternal and child health outcomes. Am J Obstet Gynecol.

[3] Deruelle P (2011). [Obesity and pregnancy]. Gynécologie Obstétrique Fertil.

[4] Strauss A, Rochow N, Kunze M, Hesse V, Dudenhausen JW, Voigt M (2021). Obesity in pregnant women: a 20-year analysis of the German experience. Eur J Clin Nutr.

[5] World Health Organization. WHO recommendations on antenatal care for a positive pregnancy experience [Internet]. 2016. 172 p. https://iris.who.int/bitstream/handle/10665/250796/9789241549912-eng.pdf?sequence=1. 28079998

[6] The American College of Obstetricians and Gynecologists. Nutrition During Pregnancy. [Internet]. 2024. https://www.acog.org/womens-health/faqs/nutrition-during-pregnancy.

[7] Xing Y, Wang X, Zhang W, Jiang H (2020). The effect of exercise on maternal complications and birth outcomes in overweight or obese pregnant women: a meta-analysis. Ann Palliat Med.

[8] Seneviratne SN, Mccowan LME, Cutfield WS, Derraik JGB, Hofman PL. Exercise in pregnancies complicated by obesity: achieving benefits and overcoming barriers. Am J Obstet Gynecol. 2014. 10.1016/j.ajog.2014.06.009 24909342

[9] Committee Opinion No (2015). 650: physical activity and Exercise during pregnancy and the Postpartum Period. Obstet Gynecol.

[10] Moyer C, Reoyo OR, May L (2016). The influence of prenatal Exercise on offspring health: a review. Clin Med Insights Womens Health.

[11] ACOG Committee Opinion No (2015). Physical activity and Exercise during pregnancy and the Postpartum Period. Obstet Gynecol.

[12] Filhol G, Bernard P, Quantin X, Espian-Marcais C, Ninot G (2014). Activité physique durant la grossesse: point sur les recommandations internationales. Gynécologie Obstétrique Fertil.

[13] Mh D, Sm R, Vj P, A JG, Ce G. N B, Prenatal exercise for the prevention of gestational diabetes mellitus and hypertensive disorders of pregnancy: a systematic review and meta-analysis. Br J Sports Med [Internet]. 2018 Nov [cited 2022 Feb 28];52(21). https://pubmed.ncbi.nlm.nih.gov/30337463/. 10.1136/bjsports-2018-099355 30337463

[14] Dodd JM, Grivell RM, Crowther CA, Robinson JS (2010). Antenatal interventions for overweight or obese pregnant women: a systematic review of randomised trials. BJOG Int J Obstet Gynaecol.

[15] Kuang J, Sun S, Ke F (2023). The effects of exercise intervention on complications and pregnancy outcomes in pregnant women with overweight or obesity: a systematic review and meta-analysis. Med (Baltim).

[16] Thangaratinam S, Rogozińska E, Jolly K, Glinkowski S, Roseboom T, Tomlinson JW et al. Effects of interventions in pregnancy on maternal weight and obstetric outcomes: meta-analysis of randomised evidence. The BMJ [Internet]. 2012 May 17 [cited 2019 Mar 18];344. https://www.ncbi.nlm.nih.gov/pmc/articles/PMC3355191/. 10.1136/bmj.e2088 PMC3355191 22596383

[17] Jacob R, Tremblay A, Fildes A, Llewellyn C, Beeken RJ, Panahi S et al. Validation of the adult eating Behaviour Questionnaire adapted for the french-speaking Canadian population. Eat Weight Disord EWD. 2021. 10.1007/s40519-021-01229-x 34185309

[18] de Lauzon B, Romon M, Deschamps V, Lafay L, Borys JM, Karlsson J (2004). The three-factor eating Questionnaire-R18 is able to distinguish among different eating patterns in a general population. J Nutr.

[19] Lesdéma A, Fromentin G, Daudin JJ, Arlotti A, Vinoy S, Tome D (2012). Characterization of the three-factor eating questionnaire scores of a young French cohort. Appetite.

[20] Chandonnet N, Saey D, Alméras N, Marc I (2012). French pregnancy physical activity Questionnaire compared with an Accelerometer Cut Point to classify physical activity among pregnant obese women. PLoS ONE.

[21] Leyrolle Q, Cserjesi R, Demeure R, Neyrinck AM, Amadieu C, Rodriguez J (2021). Microbiota and metabolite profiling as markers of Mood disorders: a cross-sectional study in obese patients. Nutrients.

[22] Bardi F, Bakker M, Kenkhuis MJA, Ranchor AV, Bakker MK, Elvan A (2021). Psychological outcomes, knowledge and preferences of pregnant women on first-trimester screening for fetal structural abnormalities: a prospective cohort study. PLoS ONE.

[23] Watson D, Clark LA, Tellegen A (1988). Development and validation of brief measures of positive and negative affect: the PANAS scales. J Pers Soc Psychol.

[24] Hanley GE, Rurak D, Lim K, Brain U, Oberlander TF (2014). The impact of maternal positive and negative affect on fetal physiology and diurnal patterns. Isr J Psychiatry Relat Sci.

[25] Sedgwick P (2015). Bias in experimental study designs: randomised controlled trials with parallel groups. BMJ.

[26] Peduzzi P, Concato J, Kemper E, Holford TR, Feinstein AR (1996). A simulation study of the number of events per variable in logistic regression analysis. J Clin Epidemiol.

[27] Bobitt J, Aguayo L, Payne L, Jansen T, Schwingel A (2019). Geographic and Social factors Associated with Chronic Disease Self-Management Program participation: going the ‘Extra-Mile’ for Disease Prevention. Prev Chronic Dis.

[28] Lin AM, Vickrey BG, Barry F, Lee ML, Ayala-Rivera M, Cheng E (2020). Factors Associated with participation in the Chronic Disease Self-Management Program: findings from the SUCCEED Trial. Stroke.

[29] Pfoh ER, Heinberg LJ, Rothberg MB (2021). Factors impacting Physician Referral To and Patient Attendance at Weight Management Programs within a large Integrated Health System. J Gen Intern Med.

[30] Porteous H, de Jersey S, Palmer M (2020). Attendance rates and characteristics of women with obesity referred to the dietitian for individual weight management advice during pregnancy. Aust N Z J Obstet Gynaecol.

[31] Piernas C, MacLean F, Aveyard P, Ahern AL, Woolston J, Boyland EJ (2020). Greater Attendance at a Community Weight Loss Programme over the first 12 weeks predicts weight loss at 2 years. Obes Facts.

[32] Haakstad LAH, Kissel I, Bø K (2021). Long-term effects of participation in a prenatal exercise intervention on body weight, body mass index, and physical activity level: a 6-year follow-up study of a randomized controlled trial. J Matern-Fetal Neonatal Med off J Eur Assoc Perinat Med Fed Asia Ocean Perinat Soc Int Soc Perinat Obstet.

[33] Guthold R, Stevens GA, Riley LM, Bull FC (2018). Worldwide trends in insufficient physical activity from 2001 to 2016: a pooled analysis of 358 population-based surveys with 1·9 million participants. Lancet Glob Health.

[34] Fielding-Singh P, Patel ML, King AC, Gardner CD (2019). Baseline Psychosocial and Demographic Factors Associated with Study Attrition and 12-Month Weight Gain in the DIETFITS Trial. Obes Silver Spring Md.

[35] Jacobson L, Wolfe M, Zackula R, Okut H, Hampton F, Grainger D et al. Electronic monitoring of Mom’s schedule (eMOMSTM): recruitment of pregnant populations with elevated BMI in a feasibility randomized controlled trial. Prev Med Rep. 2023;34. 10.1016/j.pmedr.2023.102254 PMC10244679 37292426

[36] Roche D, Rafferty A, Holden S, Killeen SL, Kennelly M, McAuliffe FM (2022). Maternal well-being and stage of Behaviour Change during pregnancy: a secondary analysis of the PEARS Randomised Controlled Trial. Int J Environ Res Public Health.

[37] Taylor C, Bhavnani V, Zasada M, Ussher M, Bick D, SWAN trial team (2020). Barriers and facilitators to uptake and retention of inner-city ethnically diverse women in a postnatal weight management intervention: a mixed-methods process evaluation within a feasibility trial in England. BMJ Open.

[38] Flannery C, McHugh S, Anaba AE, Clifford E, O’Riordan M, Kenny LC (2018). Enablers and barriers to physical activity in overweight and obese pregnant women: an analysis informed by the theoretical domains framework and COM-B model. BMC Pregnancy Childbirth.

[39] Ku CW, Leow SH, Ong LS, Erwin C, Ong I, Ng XW (2022). Developing a lifestyle intervention program for overweight or obese preconception, pregnant and postpartum women using qualitative methods. Sci Rep.

[40] Hanley S, Varley I, Sale C, Elliott-Sale K (2023). Experiences of physical activity, Healthy Eating and Quality of Life during and following pregnancy in overweight and obese Postpartum women. Matern CHILD Health J.

[41] Fair F, Soltani H (2021). A meta-review of systematic reviews of lifestyle interventions for reducing gestational weight gain in women with overweight or obesity. Obes Rev off J Int Assoc Study Obes.

[42] Barroso C, Yockey A, Degon E, Poudel P, Brown S, Hedderson M (2022). Efficacious lifestyle interventions for appropriate gestational weight gain in women with overweight or obesity set in the health care system: a scoping review. J Matern Fetal Neonatal Med.

[43] Swiss Clinical Trial Organisation. Patient and Public Involvement [Internet]. 2023. https://www.scto.ch/en/patient-and-public-involvement.html.

[44] Nagpal TS, Souza SCS, da Silva DF, Adamo KB (2021). Taking a patient-oriented approach in exercise interventions for pregnant women: a commentary. Can J Public Health.

[45] MacAulay S, Lagan BM, Casson K (2019). Planning, implementation and evaluation of antenatal weight management programmes: what are the key components? A mixed methods study. Midwifery.

[46] Gilbert A, Salvo D, Tabak R, Haire-Joshu D. Does the neighborhood built environment moderate the effectiveness of a weight-loss intervention for mothers with overweight or obesity? Findings from the healthy eating and active living taught at Home (HEALTH) study. Int J Behav Nutr Phys Act. 2022;19(1). 10.1186/s12966-022-01368-z PMC9526987 36182908

[47] Huang RC, Silva D, Beilin L, Neppe C, Mackie KE, Roffey E (2020). Feasibility of conducting an early pregnancy diet and lifestyle e-health intervention: the pregnancy Lifestyle Activity Nutrition (PLAN) project. J Dev Orig Health Dis.

[48] Ferrara A, Hedderson MM, Brown SD, Ehrlich SF, Tsai AL, Feng J (2020). A telehealth lifestyle intervention to reduce excess gestational weight gain in pregnant women with overweight or obesity (GLOW): a randomised, parallel-group, controlled trial. Lancet Diabetes Endocrinol.

[49] Greene EM, O’Brien EC, Kennelly MA, O’Brien OA, Lindsay KL, McAuliffe FM (2021). Acceptability of the pregnancy, Exercise, and Nutrition Research Study with Smartphone App Support (PEARS) and the Use of Mobile Health in a mixed lifestyle intervention by pregnant obese and overweight women: secondary analysis of a Randomized Controlled Trial. JMIR MHealth UHealth.

[50] Flannery C, Fredrix M, Olander EK, McAuliffe FM, Byrne M, Kearney PM (2019). Effectiveness of physical activity interventions for overweight and obesity during pregnancy: a systematic review of the content of behaviour change interventions. Int J Behav Nutr Phys Act.

[51] Skivington K, Matthews L, Simpson SA, Craig P, Baird J, Blazeby JM (2021). A new framework for developing and evaluating complex interventions: update of Medical Research Council guidance. BMJ.

[52] Lelorain S, Duprez C, Caton L, Nguyen MM, D’Almeida G, Cortot A (2023). Slow and steady wins the race. Lessons learned from a psychological intervention in Cancer Care: the importance of conducting a pilot and/or feasibility study in Complex interventions. Psycho-Oncol.

[53] Fontbonne A, Currie A, Tounian P, Picot MC, Foulatier O, Nedelcu M (2023). Prevalence of overweight and obesity in France: the 2020 Obepi-Roche study by the ‘Ligue Contre l’Obésité’ [League Against obesity]. J Clin Med.

