# Supplementary material for: Factors influencing participation and regular attendance in a program combining physical activity and nutritional advice for overweight and obese pregnant women
Source: BMC Pregnancy Childbirth. 2024 Jun 28;24:449. doi: 10.1186/s12884-024-06648-z (PMC11214224; doi:10.1186/s12884-024-06648-z)
Supplement: Supplementary file 1 — Supplementary Material 1 [file 12884_2024_6648_MOESM1_ESM.docx]

Additional file 1. Overview of the study


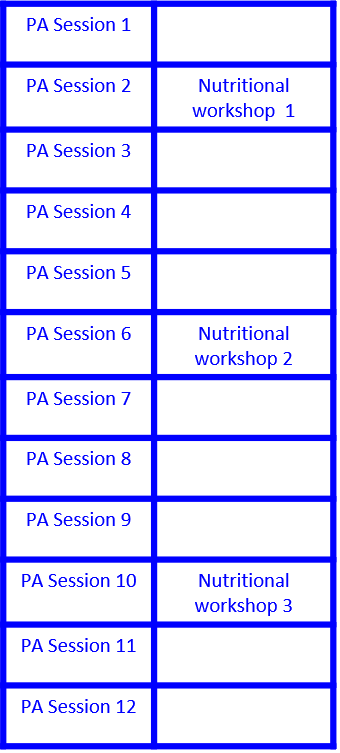


**Proposed program during 12 weeks**

**12–22^+6^ weeks**

**Identification of eligible patients**

**Proposal for participation in the "Eat well, move well for baby's health" program**

**Usual pregnancy follow-up**

One prenatal visit/month

Ultrasonography 22 SA and 32 w

Screening for gestational diabetes

and gravidic hypertension

**Second evaluation**

32**–**34 weeks

Questionnaires (PA, eating behaviors and affectivity)

**Delivery and post-partum**

Pregnancy, delivery, neonatal, and post-partum data

**During the postnatal visit 6–8 weeks after delivery**

Maternal and newborn weight

Breastfeeding duration

Questionnaires (PA, eating behaviors and affectivity)

**20–24 weeks (before program):**

- Socio-demographic data

- Clinical data

- Questionnaires (PA, eating behaviors and affectivity)

**Yes**

**No**

**Inclusion**

**Consent to be included**

PA: Physical activity
