# Supplementary material for: Factors influencing participation and regular attendance in a program combining physical activity and nutritional advice for overweight and obese pregnant women
Source: BMC Pregnancy Childbirth. 2024 Jun 28;24:449. doi: 10.1186/s12884-024-06648-z (PMC11214224; doi:10.1186/s12884-024-06648-z)
Supplement: Supplementary file 2 — Supplementary Material 2 [file 12884_2024_6648_MOESM2_ESM.docx]

Additional file 2. Description of the three nutritional workshops.

**Workshop #1** provided information on weight gain objectives during pregnancy and targeted the application of nutritional guidelines during pregnancy, according to the national health nutrition plan, or “Plan National de Nutrition Santé” (PNNS). This intervention specifically adapted the messages of the PNNS to a population of obese women through simple dietary advice, working on erroneous beliefs and representations of food, working on motivation to change eating behavior, and above all reducing the harmful link between emotions and food. In addition to these objectives, this workshop aimed to put into practice a diet adapted to pregnancy in obese women. This workshop, led by a dietician and a behavioral psychologist, took place between the 1^st^ and 4^th^ week of the program.

**Workshop #2** provided information on the benefits of breastfeeding for the baby and the mother. As in the previous workshop, the aim was to encourage the application of the PNNS dietary recommendations during breastfeeding. It tried to do this by adapting the general message to the specific problems of obese women: working on social, educational, and family difficulties, and targeting maladaptive beliefs and motivational brakes. This workshop was led by a dietician and a midwife and took place between the 5^th^ and 8^th^ week of the program.

**Workshop #3** focused on postpartum nutrition and the nutritional needs of newborns and young children. In addition to providing dietary information, special attention was given to motivating women to take care of their bodies after pregnancy and breastfeeding, and above all, to combating maladaptive beliefs about the nutritional needs of the newborn, such as those typically found among obese mothers (e.g., fear that the child will not eat enough, equating hunger with suffering, using food as an emotional substitute). This workshop took place between the 9^th^ and 12^th^ week of the program and was led by a dietician and a pediatric nurse.
